# Olive Mill Waste-Derived Activated Carbon for CO_2_ Capture Using Realistic Conditions

**DOI:** 10.1021/acs.energyfuels.4c04880

**Published:** 2025-03-07

**Authors:** Pamela B. Ramos, Arminda Mamani, María F. Sardella, Amaya Arencibia, Raúl Sanz, Eloy S. Sanz-Pérez, Marcela A. Bavio, María Erans

**Affiliations:** † Department of Chemical Engineering and Food Technology, Faculty of Engineering, UNCPBA, Avda. Del Valle 5737, Olavarría, Buenos Aires B7400JWI, Argentina; ‡ INTELYMEC. CIFICEN (CICPBA-CONICET-UNCPBA), Avda. Del Valle 5737, Olavarría, Buenos Aires B7400JWI, Argentina; § Institute of Chemical Engineering, National University of San Juan-CONICET, San Juan Av. Libertador 1109 (west), San Juan 5400, Argentina; ∥ Department of Chemical, Energy, and Mechanical Technology, ESCET, Universidad Rey Juan Carlos, C/Tulipán s/n, Móstoles, Madrid 28933, Spain; ⊥ Department of Chemical and Environmental Technology, ESCET, Universidad Rey Juan Carlos, C/Tulipán s/n, Móstoles, Madrid 28933, Spain; # Department of Chemical Engineering, Universidad de Valencia, Av. de la Universitat s/n, Burjasot, Valencia 46100, Spain

## Abstract

Olive mill waste (OMW) is a solid residue largely generated in the production of olive oil, whose haphazard dumping causes soil and water contamination due to its high content of organic compounds and characteristic acidic nature. This agroindustrial waste source can be used as a potential sustainable precursor for the production of activated carbon that can be used as an outstanding sorbent for CO_2_ capture. In this work, OMW was utilized as the activated carbon precursor, and KOH was used as the activating agent. Activation temperature, time, and KOH/carbon ratio were investigated in order to produce suitable activated carbons for CO_2_ capture. Textural and chemical characterizations were made by scanning electron microscopy (SEM), adsorption–desorption N_2_ isotherms at 77K and CO_2_ isotherms at 273 K, and Fourier transform infrared spectroscopy (FTIR). CO_2_ adsorption isotherms between 0 and 6 bar at 25 °C were obtained, and CO_2_ uptake was also measured at 30 °C in 100 mL/min of 15% vol CO_2_ for 180 min. The adsorption kinetic curves were fitted with pseudo-first-order (PFO) and pseudo-second-order (PSO) models. Also, the cyclic performance of the best adsorbent was explored for 10 adsorption/desorption cycles. The highest CO_2_ uptake was observed for the activated carbon synthesized with a KOH/precursor ratio of 2:1 and activated at 650 °C for 45 min, which had a CO_2_ uptake of 105.7 mg/g (2.4 mmol/g) in pure CO_2_ and 37.2 mg/g (0.84 mmol/g) in 15% vol CO_2_, as measured in a TGA at 30 °C.

## Graphical abstract

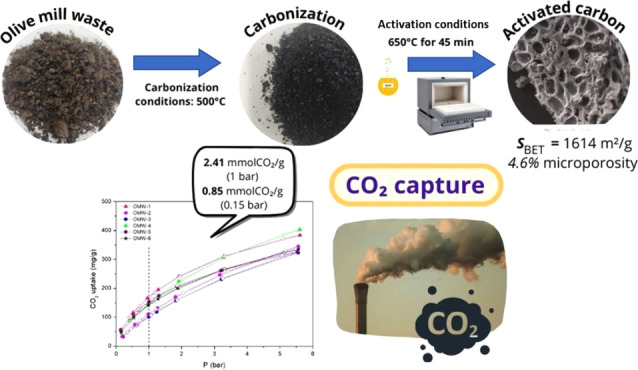

## 1 Introduction

The rising concentration of CO_2_ in the atmosphere has been causing concern in the scientific community for the last couple of decades. It should be noted that the atmospheric concentration of CO_2_ has been progressively increasing from less than 280 ppm in the preindustrial era to 420 ppm in January 2023.^1^ There is a scientific consensus that CO_2_ emissions need to be decreased by 30–60% by 2050 to maintain atmospheric CO_2_ below 550 ppm.^2^ To mitigate the effects of the current climate crisis, a mix of low-carbon technologies must be deployed. One of the many technologies that could aid in reaching this goal is carbon capture and storage (CCS).

CCS is a technology that aims at removing CO_2_ from large emission point sources such as power stations, cement, or steel industrial plants.^3^ These technologies comprise several steps, including CO_2_ capture and compression, transport, and CO_2_ storage. Different technologies have been used for CO_2_ capture; the three key strategies are (i) absorption by solvents,^4^ (ii) membrane technology,^5^ and (iii) adsorption on high surface area solids.^6,7^ Currently, the most developed technology is absorption using amine-based liquids due to their high CO_2_ uptake and industrial maturity. However, these amine solvents present their own challenges, such as high regeneration energy, equipment corrosion issues, and degradation of amines over time.^8^

As an alternative to overcoming the issues encountered when using amine solvents, numerous new materials have been synthesized to be scaled up for CO_2_ capture applications.^9–11^ Among these materials are activated carbons and carbon molecular sieves,^12,13^ mesoporous silica-based materials,^14,15^ zeolites,^16^ metal oxides,^17^ and metal–organic frameworks.^18^ Activated carbons are one of the most widely used materials in contaminant removal, such as heavy metals,^19^ dyes,^20^ pharmaceutical compounds,^21^ and volatile organic compounds,^22^ among others. Researchers are currently exploring waste materials to make these materials more environmentally friendly and move toward a circular economy scheme.^23^ The key advantage of activated carbons when dealing with CCS is the preferential adsorption of CO_2_ compared to other gases.^24^ Moreover, these adsorbents have lower sensitivity toward water as they are hydrophobic.^25^ Another advantage is their tunable chemical and physical properties via the activation process or incorporating nitrogen functionalities to increase their CO_2_ uptake.^26^

Activated carbons derived from waste materials have been obtained using a large variety of raw materials such as rice husk,^27^ nut shells,^28^ seeds,^29^ and plastics^30^ among others.^29,31,32^ One of the businesses where a large volume of waste material is generated is the agricultural industry. The residues produced by these activities, unless satisfactorily treated, could end up being incorrectly disposed of and become a serious environmental problem for both soils and water resources. These biomass sources are good candidates for activated carbon production. These solid adsorbents have been investigated for CO_2_ capture, giving different results depending on carbonization conditions, activation methodology, activating agent, temperature, and time, among others. Chemical activation with KOH has proven to be an efficient methodology for producing CO_2_ adsorbents derived from biomass.^33^ Results for CO_2_ uptake range from 1.98 mmol/g when using olive stones^34^ to 5.05 mmol/g when using black locust;^35^ both adsorption conditions were at 25 °C in pure CO_2_ at atmospheric pressure. Activated carbons as CO_2_ adsorbents have several advantages such as their lightweight, high specific surface areas, and large pore volume. Moreover, they are not moisture-sensitive, their cost is reasonable, their adsorption/desorption temperatures are low, they can be used at atmospheric pressure, and their energy consumption is low. Their key weakness is their low CO_2_ uptake at low partial pressures. Other materials such as zeolites, silica-based materials, and MOFs have a key disadvantage: their moisture adsorption and their usually complicated and expensive synthesis process.^36,37^

The olive oil industry has grown substantially in the past couple of decades. This growth has not only been limited to Mediterranean countries but also has been experienced in Serbia, Turkey, the USA, Australia, and China, among other countries.^38^ Olive oil production is an agroindustry that generates a high volume of byproducts, mainly olive mill waste (OMW) and olive mill wastewater.^39^ The introduction of the two-phase centrifugation system in the’90s in Spain reduced the production of residue in olive oil production. However, the process still generated about four million tons of solid waste annually, called olive mill waste (OMW).^40^ On average, between 70 and 80 kg of olive mill waste is generated for every 100 kg of olives.^41^

Characterization of the OMW showed a high moisture content, slightly acidic pH values, and a large content of lignin, hemicellulose, and cellulose. Different routes have been explored to reuse olive mill waste as one of the main waste sources of this industry, such as the preparation of fertilizers. However, its addition to soil has proven to have a detrimental effect on seed germination, plant growth, and microbial activity.^40^

OMW could be a potential biomass precursor for activated carbons (AC) for CO_2_ capture.^42^ Different methods have been investigated for activated carbon derived from olive waste, such as physical activation using CO_2_, chemical activation using KOH, and doping with N-functional groups. Maximum CO_2_ uptakes of 3.521 mmol/g and 2.984 mmol/g were reported for the N-doped material, and the KOH-activated AC, respectively, at 0 °C in pure CO_2_ conditions.^41^ Olive waste has also been used as a precursor using H_3_PO_4_ and ZnCl_2_ as activating agents; the activated carbon produced had a CO_2_ uptake of 18.6 mg/g at 0.1 MPa.^43^

In this work, olive mill waste (OMW) was used to produce activated carbons chemically with KOH. Three variables were studied: (i) KOH/biochar ratio, (ii) activation temperature, and (iii) activation time. All of these adsorbents were characterized thoroughly to gain an understanding of their physical and chemical properties. Then, their CO_2_ uptake was investigated through isothermal and kinetic analyses under a variety of conditions. This work focuses on testing these materials under realistic CO_2_ concentrations in order to assess their suitability for deploying industrial CO_2_ capture systems as opposed to pure CO_2_ as the reacting gas. Finally, their cyclic capacity was also studied to investigate the feasibility of the capture/regeneration operation.

## 2 Experimental Section

### 2.1 Materials

Olive mill waste (OMW) was employed to produce activated carbons (AC). This waste was collected from the olive oil production process of the company EL MISTOL S.A., located in the Cuyo region (Argentina). For chemical activation, KOH (potassium hydroxide pellets ≥85%) and HCl (37 wt %) from Sigma-Aldrich were used. The gases used in this work were purchased from Air Liquide, CO_2_ N38 with a purity of 99.99% N_2_, and a mixture of 15% CO_2_ and 85% N_2_. Milli-Q grade distilled water was used to wash and neutralize the activated carbons.

### 2.2 Activated Carbon Synthesis

The waste samples were collected and dried in an oven at 105 °C. The first step, carbonization, was carried out in an electrically heated tube furnace under an inert atmosphere. The carbonization temperature was set to 500 °C with a heating rate of 4 °C min^–1^ and a heating time of 120 min. After the carbonization process was finished, the particle size was brought to a granulometry between 4 and 18 ASTM mesh.

The carbonized samples were then chemically activated using an 80% w/v KOH solution. Different KOH/biochar ratios were used: 2, 4, and 6 g per g. Moreover, different activation temperatures (650 and 950 °C) and activation times (45, 75, and 120 min) were explored. The activation process was performed as follows: a dispersion was prepared with the biochar and activating agent, and the mixture was stirred for 15 min at room temperature. Then, it was dried at 110 °C in an oven for 24 h. The dried sample was transferred to a stainless steel reactor with electrical heating without oxygen until the desired temperature for a time set. The obtained activated carbon was cooled and washed with 0.1 M HCl, and deionized water until the filtrate reached a neutral pH. The samples were denoted with the following nomenclature, OMW-X, where X is the sample number. The samples used in this work are listed in Table 1 with their activation conditions.

**Table 1:** Activation Conditions for OMW Carbons

Sample name	KOH/Biochar ratio (g/g)	Activation temperature (°C)	Activation time (min)
**OMW-1**	2	650	45
**OMW-2**	2	950	45
**OMW-3**	2	950	120
**OMW-4**	4	650	75
**OMW-5**	4	650	120
**OMW-6**	6	650	75

### 2.3 Material Characterization

The moisture content of the OMW was determined using the ASTM D-4442 standard. The determination of cellulose, hemicellulose, and lignin was conducted using the TAPPI^44^ and ASTM standards (ASTM 1106). Ultimate analysis was performed in an (CHNS-O EA1108) elemental analyzer as defined by the CEN/TS 15,104 standard.

The characterization of the OMW and activated carbons using physicochemical techniques was carried out. The selected carbonization temperature was evaluated by thermogravimetric analysis of the OMW biomass. It was performed in an SDT Q600 analyzer with a temperature range of 22–900 °C and a heating rate of 10 °C min^–1^, under nitrogen flow. Previously, the OMW sample was dried at 105 °C according to ASTM standards. The morphology of the activated carbons was analyzed using an SEM-LEO-EVO 40 XVP microscope. Functional groups were analyzed by Fourier transform infrared spectroscopy (FTIR) using a Nicolet-Magna 550 spectrometer with CsI optics on KBr disks. Nitrogen adsorption–desorption isotherms at 77 K characterized activated carbons measured in a Micromeritics Gemini V2.0 2380; samples were degassed at a constant temperature of 250 °C for 12 h in Micromeritics FlowPrep 060 equipment. The Brunauer–Emmett–Teller (BET) model calculated the specific surface area. The Gurvich’s rule at P/Po = 0.98 and the Dubinin–Radushkevich’s model (w_0_) determined the micropore and total pore volumes, respectively. In order to determine the narrow micropore pore size distributions, CO_2_ adsorption isotherms at 273 K were determined using a Micromeritics Triflex system. Pore size distribution was calculated by applying the non-local density functional theory (NLDFT) using the heterogeneous model for carbons.

### 2.4 CO_2_ Capture Experiments

CO_2_ isotherms between 0 and 6 bar were obtained using volumetric adsorption equipment (HPVA-100, VTI Scientific Instruments) at 25 °C. Previously, all the samples were degassed for 2 h at 110 °C under vacuum. Two combined equilibrium criteria were used for isotherm obtention: (i) pressure drop below 0.2 mbar for 3 min or (ii) 50 min of equilibration time.

The CO_2_ adsorption kinetics were measured using a TGA/DSC 1 STARe System from Mettler Toledo. CO_2_ uptake was measured at 30 °C in 100 mL/min of 15% vol CO_2_ in N_2_ and 100% CO_2_ for 180 min. Previously, the samples (ca. 10 mg) were degassed for 2 h at 110 °C in 100 mL/min of pure N_2_.

Pseudo first-order (PFO) and pseudo second-order (PSO) kinetic models were applied to the experimental measurements to analyze the adsorption kinetics and study the CO_2_ adsorption process on activated carbons (see Supporting Information, SI-1).^45^

Moreover, the cyclic performance of the best adsorbent was explored. The samples were degassed for 2 h at 110 °C in 100 mL/min of N_2_. Then, the temperature was reduced to 30 °C in 100 mL/min of N_2_, and they were put in contact with pure and 15% vol CO_2_ for 25 min. After the adsorption step, the sample was heated again to 110 °C under 100 mL/min of N_2_ and degassed between adsorption cycles. These steps were repeated for 10 adsorption/desorption cycles.

## 3 Results

### 3.1 Biomass and Activated Carbon Characterization

Table 2 shows the proximate and ultimate analyses and the cellulose, hemicellulose, and lignin contents of the olive mill waste.

**Table 2:** Chemical Composition of the Olive Mill Waste (OMW)

Parameter	Value (% dry basis)
Moisture content (%wt)	67.0 ± 0.12
Ash	7.2 ± 0.7
Volatile matter	15.0 ± 0.4
Fixed carbon (%wt)	10.79 ± 0.21
Cellulose (%wt)	30.19 ± 0.15
Hemicellulose (%wt)	15.64 ± 0.14
Lignin (%wt)	51.7 ± 0.4
C (%wt)	51
H (%wt)	7.5
O (%wt)	31.4
N (%wt)	0.92

The results show a high moisture content (67.0 ± 0.12 wt %) due to the nature of the biomass. It is necessary to condition it through a drying and storage process before using it in the synthesis of activated carbons. Low ash (7.18%) and volatile matter (15%) contents are desirable for good porous development and mass yield, making the mill olive residue an excellent precursor for synthesizing activated carbons. Similar results were reported by Navas et al.^46^ for the olive pit residue, where the values of ash and fixed carbon were 6.79% and 11.66%, respectively. However, a significant difference in volatile matter (77.28%) is observed due to the nature of the residue. González-García^47^ carried out a proximal composition analysis of various biomasses used for activated carbon synthesis. The report for the barley husk residue (6.7% ash and 18.1% fixed carbon) and for the tomato stems (10.6% ash) was similar to that obtained in this work for OMW.

Additionally, the lignin, cellulose, and hemicellulose for the OMW were determined, where the results show that the residue has a high content of cellulose (30.19%), a high content of hemicellulose (15.6%), and lignin (51.7%), giving result values similar to those reported by Demiral et al.^48^ for another type of biomass. Olive bagasse has a cellulose content of 31.1% and hemicellulose of 15.6%, in agreement with the values obtained for OMW; however, the amount of lignin is double in OMW compared to that reported for olive bagasse due to the nature of the residue. In addition, the contents of C, O, H, and N reported for olive bagasse are 53.4%, 7.5%, 37.4%, and 1.7% for C, O, H, and N, respectively. The high C, O, and H contents are directly related to the cellulose, lignin, and hemicellulose in the olive mill residue and most lignocellulosic precursors used to synthesize activated carbons.

The behavior of the olive mill waste (OMW) using a thermogravimetric analysis (TGA) was studied. Figure 1a shows the thermogravimetric curve from 25 to 900 °C, with a total mass loss of 85.94%. Three stages of decomposition are observed. The first, up to 100 °C, is related to the loss of moisture from the residue. Then, in the second stage, between 250 and 456 °C, a sharp decrease in mass is observed due to pyrolysis and degradation/oxidation reactions of cellulose and xylose. The third stage begins at 456 °C, corresponding to the slow degradation of lignin and cellulose decomposition. In the second stage, there is a mass loss of 65.47% and a mass loss of 12.87% in the third stage, similar to that obtained elsewhere.^49^ Therefore, with the TGA analysis, it was possible to select the carbonization temperature of the olive oil residue at 500 °C.^49^

**Figure 1:**
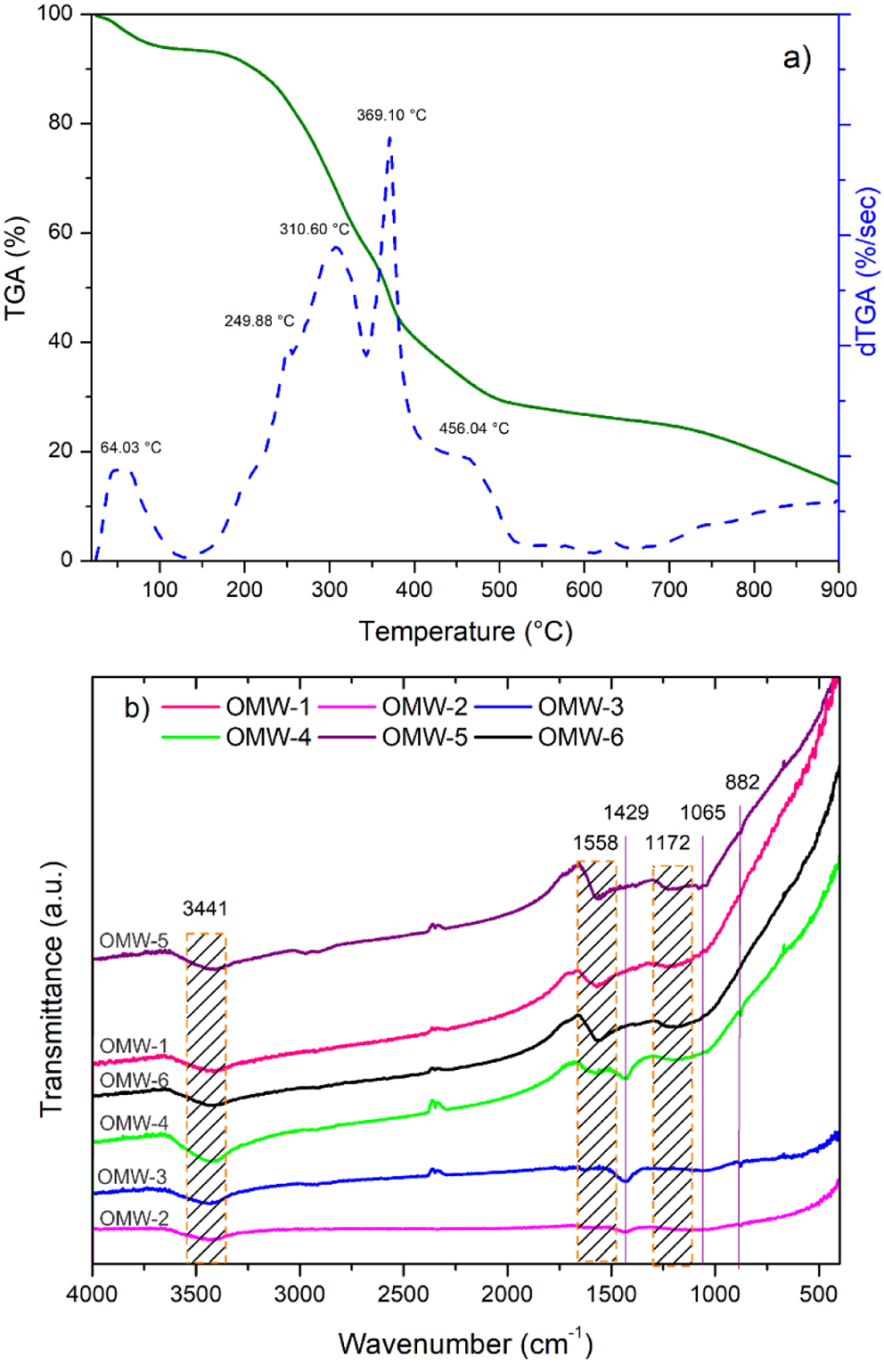
(a) TGA analysis of olive mill waste performed under N_2_ and (b) FT-IR spectra of activated carbons from olive mill waste.

The FTIR spectra for the activated carbons from the OMW are shown in Figure 1b. Activated carbons under different conditions show a band associated with the presence of −OH bonds and chemisorbed water at 3441 cm^–1^. In turn, it shows a band between 1600 and 1500 cm^–1^ associated with the stretching of C–O and aromatic structure, and a band around 1430–1100 cm^–1^ is due to the O–H and C–H bending vibrations. The peaks at 880 and 1065 cm^–1^, present in the activated carbons OMW3 and OMW5, are associated with the C–O stretch. A decrease in the peak intensity associated with the C=C bonds in the lignin (1558 cm^–1^) is evident in the activated carbons at a higher activation temperature (OMW-2 and OMW-3). As well as with the increase in the activation temperature, a decrease in the content of oxygenated groups in the activated carbons can be observed (C=O stretching vibration of carbonyl, carboxyl, and acetyl groups, bond for 1730–1640 cm^–1^)

SEM was used to evaluate the morphologies of the surfaces of the different activated carbons. Figure 2 shows that activated carbons present different porous structures that directly depend on the activation time, activation temperature, and KOH/biochar ratio.

**Figure 2:**
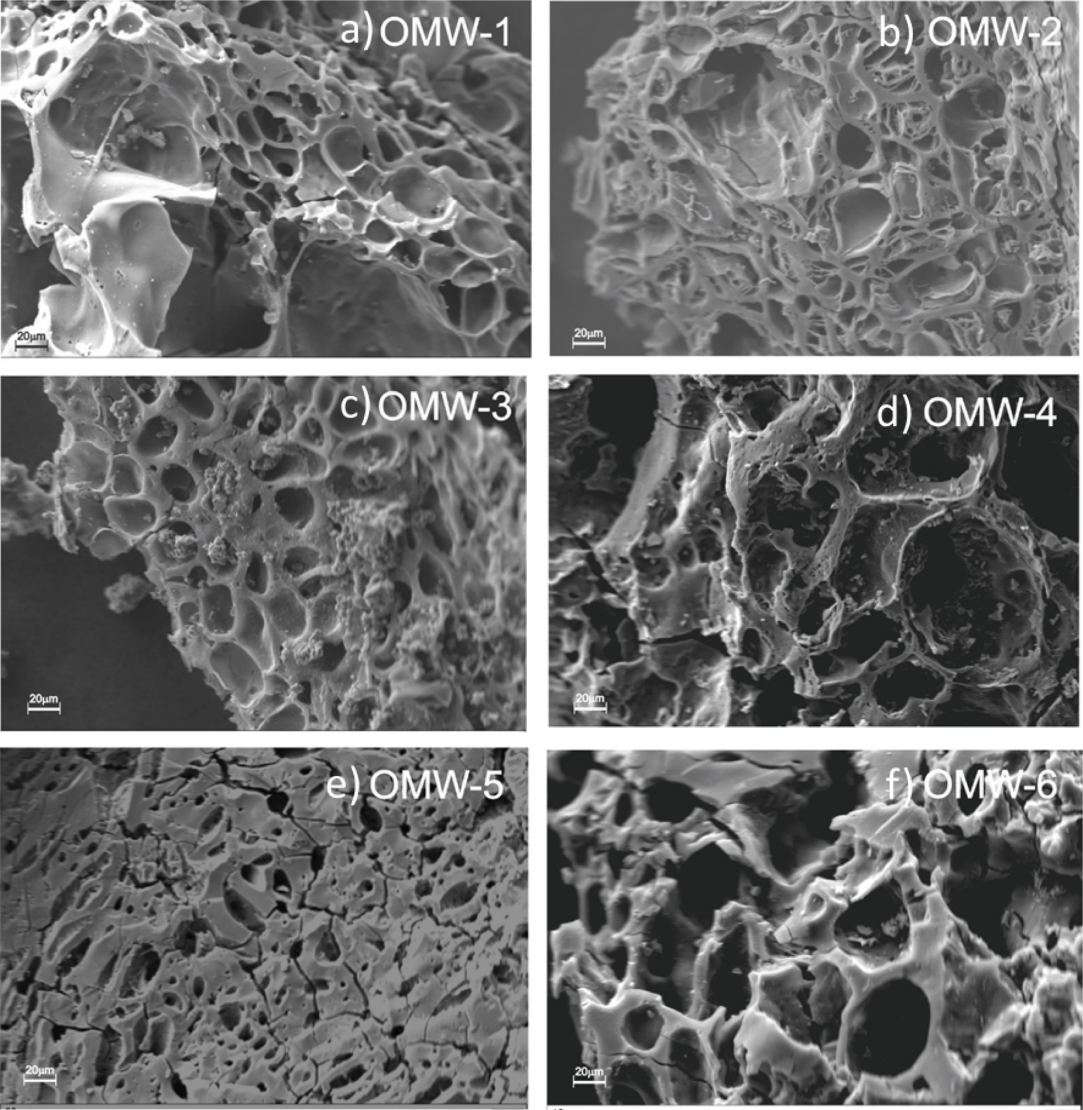
SEM micrographs at 1000 × of activated carbons: (a) OMW-1, (b) OMW-2, (c) OMW-3, (d) OMW-4, (e) OMW-5, and (f) OMW-6.

The surface of the activated carbons is irregular, with holes of different sizes, and no channel-shaped cavities are observed. Figure 2a,b shows that an increase in the activation temperature promotes larger and nonuniform cavities (various sizes) due to the greater degradation of cellulose, hemicellulose, and lignin performed at high temperatures.^50,51^ Analyzing Figure 2b,c and Figure 2d,e, it is observed that an increase in the activation time causes the development of holes of more regular sizes and generates cracks on the carbon surface (observed in Figure 2e). Regarding the variation of the impregnation ratio, an increase from 4/1 to 6/1 produces a defined porous structure and presents a more significant number of small holes (Figure 2d,f).

Figure 3 shows the nitrogen adsorption–desorption isotherms at 77K for the AC samples. Activated carbons present type I (IUPAC) isotherms typical of microporous materials and type IV corresponding to mesoporous materials.^47^ The behavior of the type I isotherm is associated with monolayer adsorption, where the amount of N_2_ adsorbed increases significantly at P/P0 < 0.1 and more slightly in the range of P/P_0_ > 0.1, which indicates the predominance of the microporous structure in activated carbons. OMW-1 and OMW-2 exhibit an isotherm with a small hysteresis loop, which means that a certain number of mesopores are present (low ratio). However, the horizontal branch of saturation (P/P_0_ > 0.4) suggests the preponderance of micropores over mesopores. Isotherms of the activated carbon OMW-3 (type IV) present a well-defined hysteresis loop, and at pressures greater than 0.4, the saturation branch is not observed.

**Figure 3:**
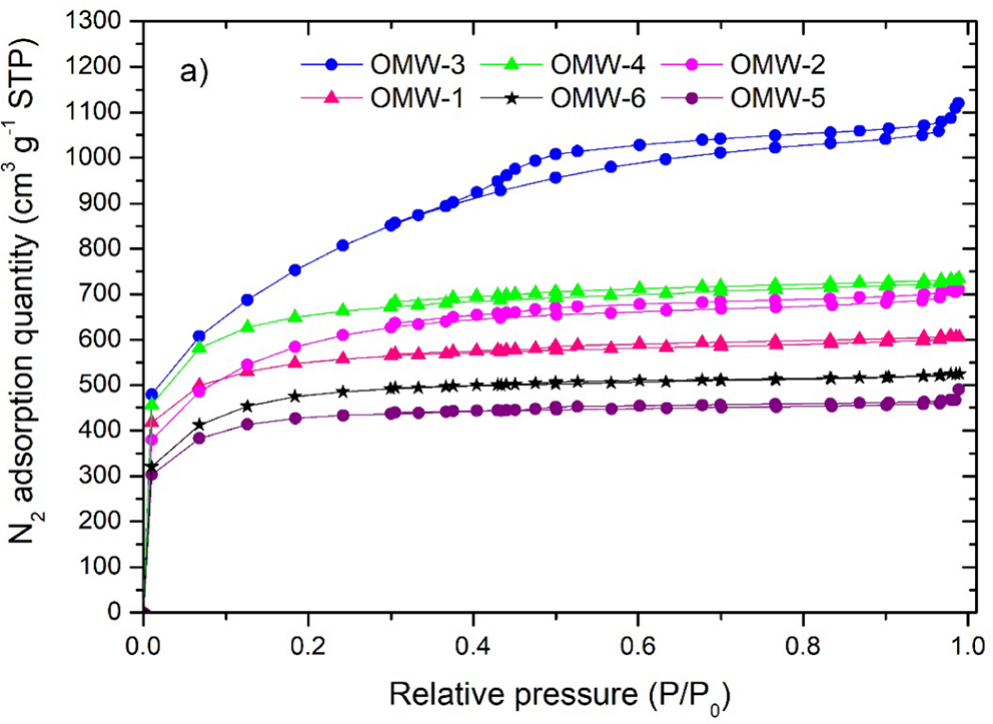
Nitrogen adsorption–desorption isotherms of all of the prepared activated carbons.

Table 3 shows the textural properties such as pore size, surface area (*S*_BET_), volumes of pores and micropores, the percentage of micropores, and mass yield (%). It is observed that from the activation process applied to the oil mill waste, porous structures are developed with surface areas of the order of 1251 m^2^g^–1^ and 2577 m^2^g^–1^ and a total pore volume of 2.29 cm^3^ g^–1^ and 0.76 cm^3^ g^–1^. The activated carbon OMW-3 presents an *S*_BET_ of 2577 m^2^g^–1^ greater than those of the other activated carbons. It is observed that most of the activated carbons have a high microporous volume. The lowest surface area was obtained at an activation temperature of 650 °C, an activation time of 120 min, and a KOH/biochar of 4/1 g g^–1^ (OMW-5).

**Table 3:** Textural Properties of Activated Carbons

Sample name	Pore size^a^ (Å)	S_BET_ (m^2^g^–1^)	V_total_ (cm^3^g^–1^)	V_micro_ (cm^3^g^–1^)	V_micro_/V_total_ (%)	Mass yield^b^ (%)
OMW-1	4.8	1614	0.94	0.89	94.6	92.3
OMW-2	5.6	1884	1.09	0.98	89.9	74.3
	5.7					
OMW-3	5.9	2577	2.29	1.31	57.2	64
OMW-4	5.3	1932	1.14	1.07	93.8	68
OMW-5	5.1	1251	0.76	0.69	90.7	68.5
OMW-6	4.8	1418	0.81	0.78	96.3	58.2

a Calculated from the CO_2_ isotherms at 273 K.

b The mass yield (%) of activated carbons has been calculated from the carbonized material.

An increase in the activation time (from 45 to 120 min) causes an increase in the total pore volume and micropore volume in the carbon structure for an impregnation ratio of 2/1 g g^–1^ (see Table 3), but there is a decrease in the percentage of microporosity (from 89.9% to 57.2%). However, for an impregnation ratio of 4/1 g g^–1^ (OMW-4 and OMW-5), a decrease in the surface area and pore volume is observed, which could be caused by the collapse of the material structure.^52^ The relationship between V_micro_ and *V*_total_ (% micropore) and the pore size decreases with increasing activation time.

However, the activated carbon OMW-3 has the lowest micropore content; specifically, 57.2% of the total pore volume is micropores. Comparing the activation conditions used for the preparation of the activated carbons, it is observed that an increase in the activation temperature from 650 to 950 °C causes an increment in the total pore volume and the micropore volume with an increment of surface area (comparison between OMW-1 and OMW-2). The rise in activation time (OMW-2 and OMW-3) leads to carbon with higher surface and pore volume, although reduced percentage of V_micro_/*V*_total_ present in the carbon structure. On the other hand, an increase in the impregnation ratio (comparison between OMW-4, and OMW-6) generates a low total pore volume, but the micropores (%) developed is more significant (96.3%) for OMW-6). It has been shown that high impregnation ratios of KOH produce more micropore development in the activation process.^53^ The increase in activation temperature and impregnation ratio affects the mass yield of activated carbons, causing greater degradation and oxidation of hemicellulose and cellulose and consequently a decrease in the mass yield (%) of the materials.

### 3.2 CO_2_ Capture

CO_2_ adsorption isotherms for the different activated carbons derived from the OMW can be seen in Figure 4. All of the synthesized materials present significant CO_2_ uptakes at all pressure levels. The sharp surge in CO_2_ uptake with increasing pressure and the reversibility of the adsorption and desorption branches are characteristics of physical adsorption processes, as expected.^54^ All activated carbons lack a hysteresis loop, which is also typical of the physical adsorption of carbon atoms in CO_2_. The measured values for CO_2_ adsorbed at 1 bar using this volumetric method are between 166 mg/g for OMW-1 and 102 mg/g for OMW-2.

**Figure 4:**
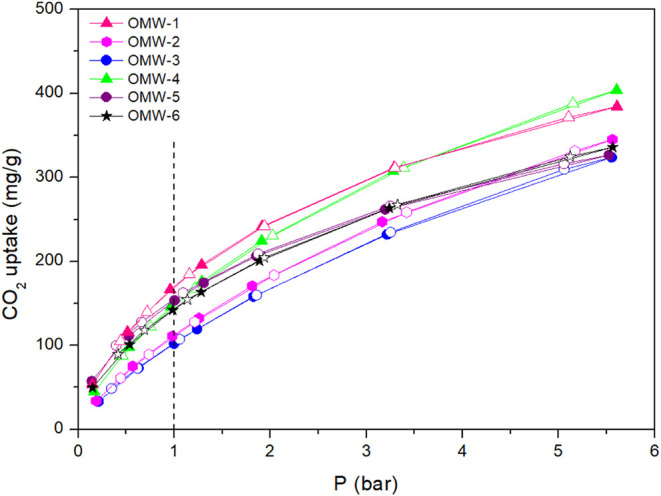
CO_2_ isotherms for olive mill-waste-derived activated carbons at 25 °C.

At low pressures (*p* < 0.2 bar), the CO_2_ uptake values for all activated carbons are from 25 to 50 mg/g approximately, while at high pressures of CO_2_ (5 bar), there is a noteworthy variance in the adsorption values, with OMW-4 having the best result. The performance at high pressure (CO_2_ uptake of 404 mg/g at 5.6 bar) can be explained by the higher micropore percentage found for this material (1.07 cm^3^/g). However, the OMW-3 sample showed a higher value of micropore volume and no higher adsorption of CO_2_ was found.

To investigate the results in depth, the CO_2_ isotherms of the activated carbons at 273 K were measured, and the micropore size distributions have been obtained from these CO_2_. The data are presented in Figure 5, and the average pore sizes are included in Table 3. In the pore size distribution, it can be seen how samples OMW-1, OMW-5, and OMW-6 have a higher proportion of very narrow pores with sizes of 4.8, 5.1, and 4.8 nm, respectively. These narrow micropore sizes are responsible for CO_2_ adsorption at atmospheric conditions.^55^ On the other hand, the PSD distribution was found to be much wider for the samples OMW-2 and OMW-3 (average sizes of 5.6 and 5.9 A, respectively), showing a lower proportion of narrow pores and a higher contribution at larger sizes. Although the larger pores could increase the adsorption capacity of CO_2_, as in the case of OMW-4, the smaller amount of narrow pores should be dominant and, thus, the CO_2_ adsorption capacity of samples OMW-2 and OMW-3 is lower.

**Figure 5:**
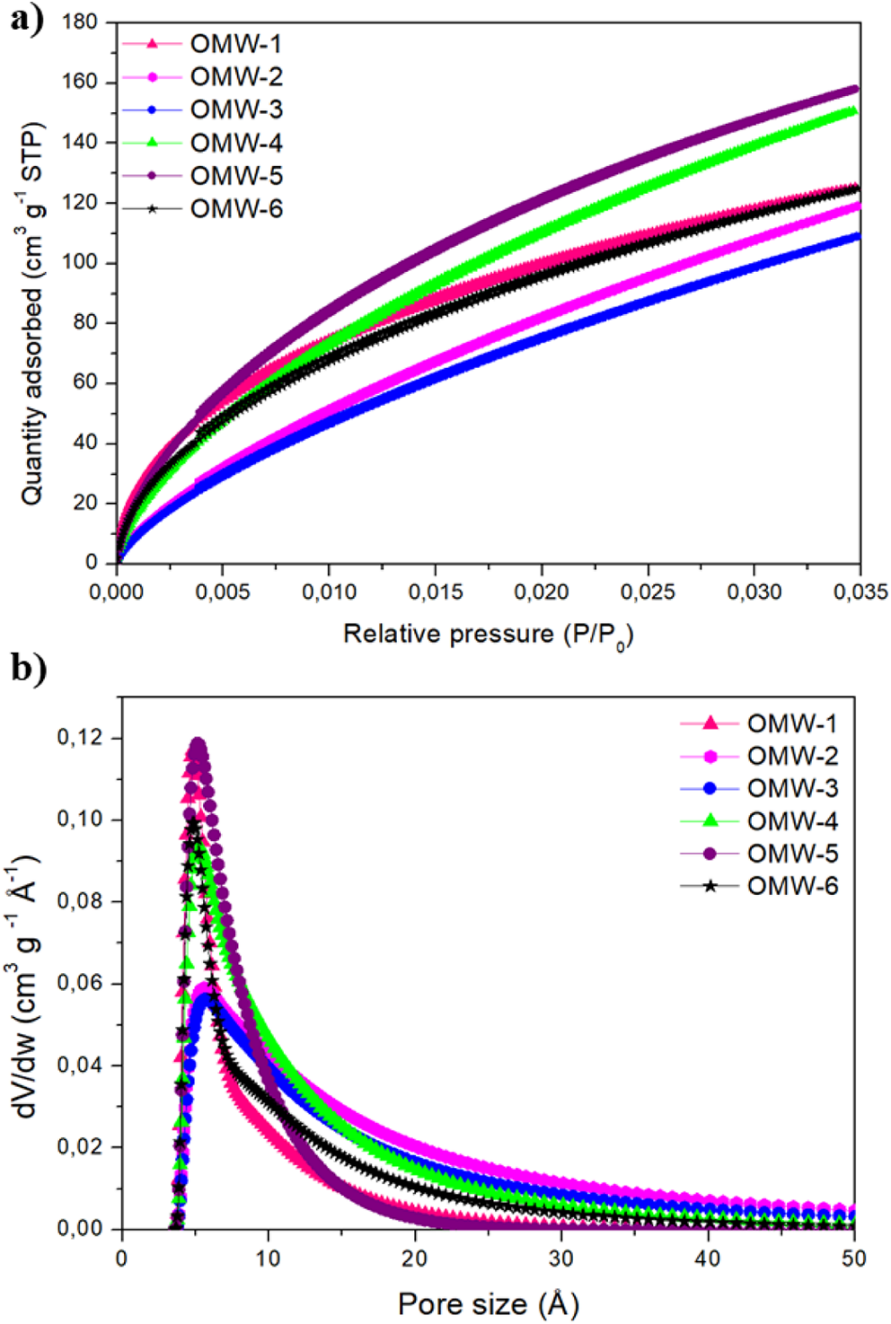
(a) CO_2_ adsorption isotherms at 273 K and (b) pore size distributions of the prepared activated carbons.

These results also show the effect of the studied parameters on measured CO_2_ uptake. It can be seen that the CO_2_ uptake decreases when increasing the activation temperature as it happens with OMW-1 and OMW-2, where this fact is observed in the entire pressure range. Total pore volume and microporosity were increased when the temperature was augmented from 650 to 950 °C in OMW-1 and OMW-2, proving that rising the temperature is unfavorable for this application because of the decreased ratio V_micro_/V_total_ (%). Another interesting point is the increase of surface area, porosity, and micropore volume with longer activation treatments while decreasing the mean pore diameter for an impregnation ratio of 2/1. Additionally, this trend is noted at different temperatures and KOH/biochar ratios with OMW-2 and with OMW-3, OMW-4, and OMW-5. The percentage of microporosity increases when the KOH/biochar ratio is risen,^56^ namely from 93.8 to 96.3% in OMW-4 and OMW-6, respectively, while total porosity and surface area decrease. It can be inferred that lower activation temperatures result in higher CO_2_ uptake, as observed in OMW-2 versus OMW-1. Activation time variation seems to have a less significant role, as seen in OMW-3 and OMW-2, and also in OMW-4 and OMW-5, where these pairs have similar CO_2_ uptakes regardless of activation time. KOH/biochar also has an impact on CO_2_ uptake, with increasing CO_2_ uptake with decreasing KOH/biochar ratio, as is depicted in OMW-4 and OMW-6.

In real processes, the composition of flue gas ranges between the following values: 8–15% CO_2_, 8–20% H_2_O, and 2–5% O_2_.^57^ Therefore, a value of 15 vol % CO_2_ is more representative of what is expected in postcombustion applications.

Figure 6a,b shows the CO_2_ uptake obtained by thermogravimetric analysis (TGA) for a 15 vol % CO_2_/N_2_ mixture, a representative concentration for a typical coal-fired power plant, and pure CO_2_, respectively. The adsorption behavior of the activated carbons when tested with 100% CO_2_ at 1 bar (both by volumetric methods and by thermogravimetric analysis) has a similar trend and adsorption capacity. The carbons are ranked in order from highest to lowest adsorption capacity as follows: OMW-1 > OMW-5 > OMW-4 > OMW-6 > OMW-2 > OMW-3. However, the behavior of the activated carbons for a 15% CO_2_/N_2_ mixture (Figure 6a) differs from the trend observed for 100% CO_2_ at 1 bar (Figure 6b). The values obtained at this lower partial CO_2_ pressure are in the range of 25% to 35% of those measured at 1 bar. The best-performing material, OMW-1, has 35% of the CO_2_ uptake calculated in pure CO_2_ at 30 °C. These observed high values at low partial pressure are a good indicator of the opportunities for these olive mill waste KOH-activated carbons as CO_2_ adsorbents in different applications such as industrial plants, biogas cleaning, and decarbonization of coal. Olive mill waste has been investigated before for CO_2_ capture with different physical and chemical activation procedures. However, biochar was obtained via hydrothermal methods as opposed to pyrolysis. Nonetheless, it was found that the best materials synthesized with KOH were activated at 600 °C with a KOH/biochar ratio of 5 and an activation time of 30 min. The isotherms for this material revealed a CO_2_ uptake at 25 °C of 50.6 mg/g at 0.15 bar^42^ compared to OMW-1 and OMW-5 with 53.3 mg/g (1.21 mmol/g) and 57.3 mg/g (1.3 mmol/g), which is a 5 and 13% increase, respectively, in the same isotherm conditions.

**Figure 6:**
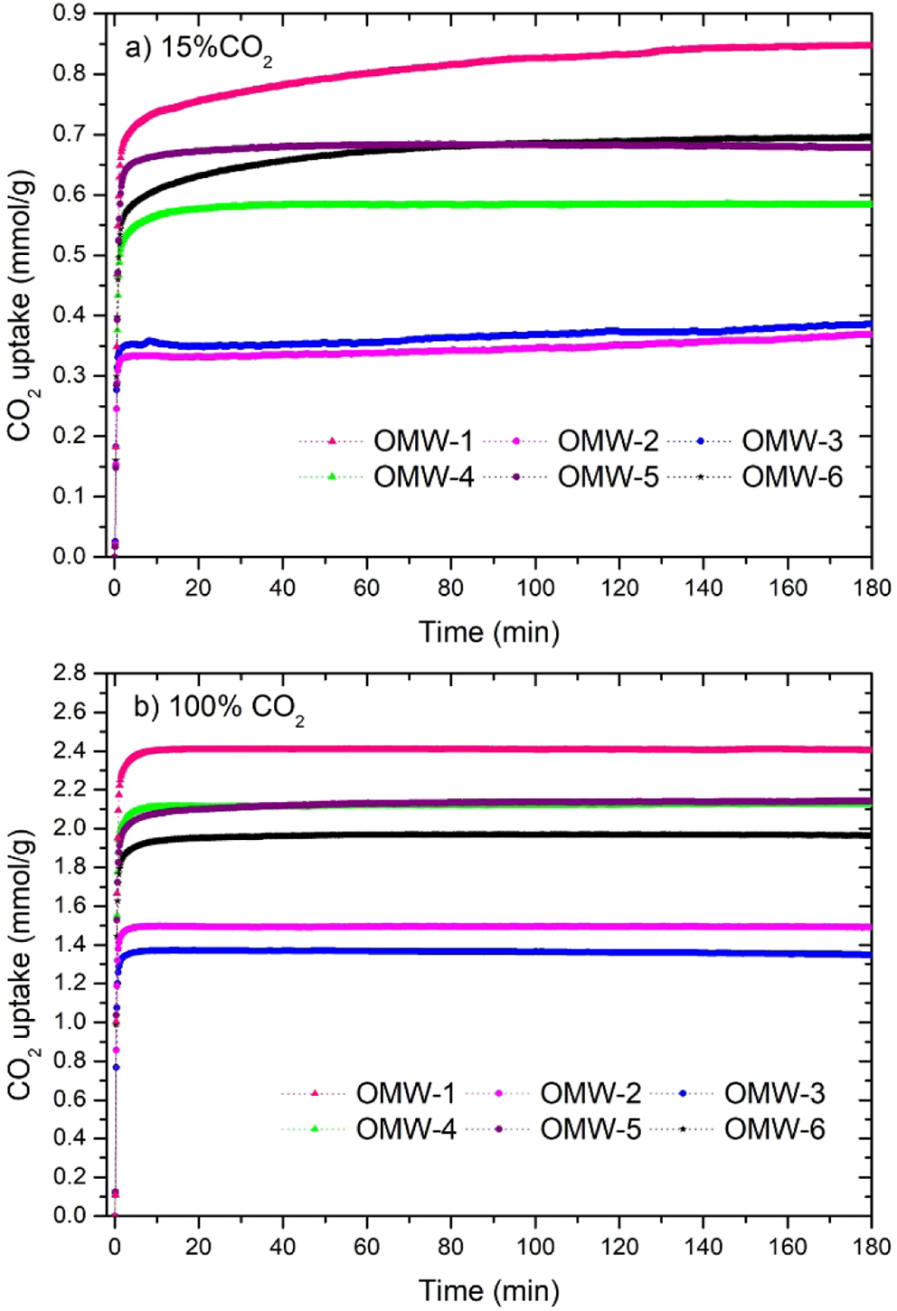
CO_2_ uptake of OMW activated carbons at 30 °C using (a) 15% CO_2_/N_2_ mixture and (b) 100% CO_2_.

Some values at 0.15 bar and 25 °C of chemically activated carbons produced from different precursors are given in Table 4 for comparison purposes.

**Table 4:** CO_2_ Uptake at 0.15 Bar and 25 °C of Carbons Chemically Activated with KOH

Precursor	CO_2_ uptake (mg/g)	CO_2_ uptake (mmol/g)	Reference
Olive mill waste	57.3	1.30	This work
Olive mill waste (hydrothermal char)	50.6	1.15	^42^
Rice husk	66.4	1.51	^59^
Coconut shell	61.6	1.40	^60,61^
Camphor leaves	48.6	1.10	^62^
Pomegranate peels	55.0	1.25	^63^

The results shown above corroborate the suitability of this precursor and activating method as a CO_2_ adsorbent for combustion applications, especially in areas where olive oil is produced, where this waste stream is widely available and cheap.

To select the best-performing activated carbon for CO_2_ capture and subsequent reuse cycles, it is very important to know the kinetics and model of the CO_2_ adsorption process. Pseudo-first-order (PFO) and pseudo-second-order (PSO) models were used to estimate the parameters and kinetic characteristics of CO_2_ adsorption on activated carbons. The experimental data of CO_2_ adsorption capacity as a function of time were fitted to the pseudo-first-order (PFO) kinetic model, which is the best fit for the experimental data, resulting in high correlation coefficients (R^2^ > 0.989) for 15% CO_2_ and 100% CO_2_, which shows that the PFO model adequately fits the experimental data for activated carbons. Figure 7a shows that the different carbons have fast kinetics; for an adsorption time of 2 min, the materials reached approximately 92% of the CO_2_ adsorption in equilibrium. Table SI-1.1 shows the high correlation coefficient values of R^2^ > 0.989 for all samples. This model predicts the adsorption behavior of CO_2_ on physical adsorbents and represents a reversible interaction between CO_2_ molecules and activated carbons.

**Figure 7:**
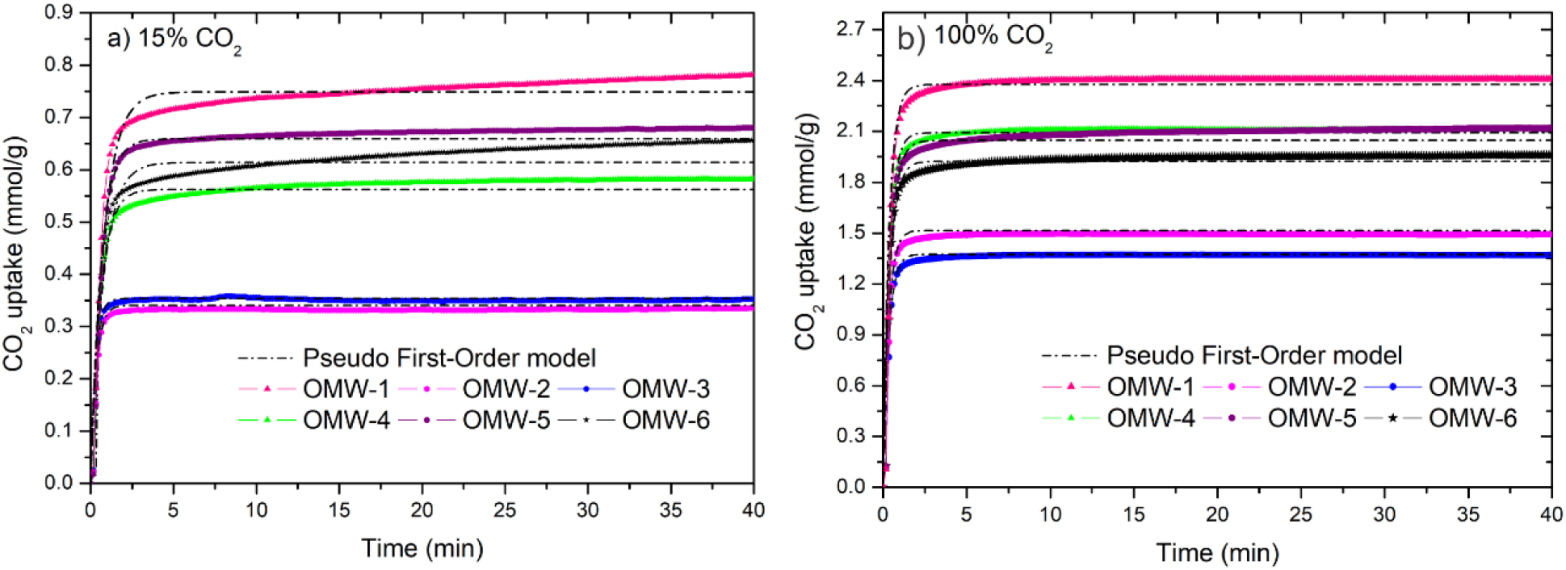
Experimental and predicted CO_2_ adsorption capacities by pseudo first-order (PFO): (a) 15% CO_2_/N_2_ mixture and (b) 100% CO_2_.

Tables SI-1.1 and SI-1.2 show the kinetic parameters of CO_2_ adsorption obtained from the pseudo-first-order model fitting. The correlation coefficients and normalized standard deviation, calculated from eq SI-1.3, are also shown. For the pseudo-first-order model, high kinetic constant values were obtained due to the fast adsorption kinetics observed experimentally. For all activated carbon samples, the kinetic constants (KF) increase when the CO_2_ concentration increases from 15% vol CO_2_ to 100% CO_2_; this trend is similar to that observed in the work performed by Álvarez-Gutiérrez et al.^58^

The cyclic capacity of the OMW-1 was investigated with a flow of pure CO_2_ at an adsorption temperature of 30 °C and is shown in Figure 8. The CO_2_ uptake after each cycle had a minor reduction, with a total of 1.8% loss after 10 cycles. This is related to the adsorption mechanism as physical adsorption dominates the process, with the interactions between the adsorbent and the CO_2_ being weak. Other materials typically used for CO_2_ capture, such as amine-functionalized adsorbents, have higher CO_2_ uptake as chemical adsorption dominates the reaction mechanism.^64^ However, their reactivity decay is higher in adsorption–desorption cycles, with typical losses ranging from 4% to 14% after 10 cycles.^65–67^

**Figure 8:**
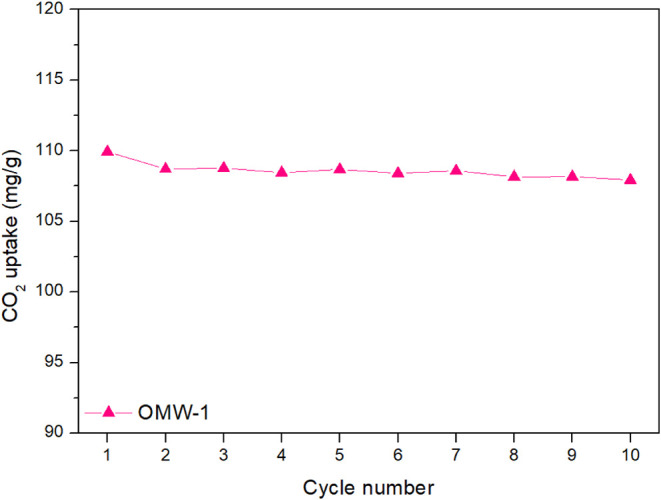
Cyclic CO_2_ uptake of syringe of OMW-1 at 30 °C.

## 4 Conclusions

The results of this study demonstrate that olive mill waste-derived activated carbon synthesized with KOH activation provides a wide range of CO_2_ capture performance, depending on the activation conditions. It was found that the increase of the impregnation ratio yields a lower total pore volume, although the ratio of micropores to total pore volume was higher. Besides, a decrease in the level of CO_2_ uptake in the entire pressure range was detected when the activation temperature increased. On the other hand, the treatment time seems to have little effect in terms of CO_2_ capture at the two studied temperatures and KOH ratios.

The highest CO_2_ uptake was observed for the OMW-1 activated carbon synthesized with a KOH/precursor ratio of 2:1 and activated at 650 °C for 45 min, which showed a CO_2_ uptake of 105.7 mg/g (2.4 mmol/g) in pure CO_2_ and 37.2 mg/g (0.84 mmol/g) in 15% CO_2_. The activated carbon synthesized from olive mill waste exhibited a relatively high mesopore volume, which may have contributed to the CO_2_ capture performance. The activated carbons presented fast adsorption kinetics and pseudo-first-order (PFO) behavior. This study highlights the importance of controlling mesoporosity to optimize the CO_2_ capture potential of activated carbons. Moreover, OMW-1 was found to be highly stable when used in adsorption–desorption cycles, with only 1.8% loss after 10 cycles in pure CO_2_.

The findings of this study have significant implications for the development of efficient and sustainable materials for CO_2_ capture. The utilization of olive mill waste as a precursor material for activated carbon synthesis presents an opportunity for the valorization of agricultural waste. Additionally, optimization of the activation conditions can lead to significant improvements in the CO_2_ capture performance of the activated carbons. The results of this study can contribute to the development of effective strategies for mitigating climate change by reducing CO_2_ emissions by using activated carbons for CO_2_ capture. These materials could be implemented at large scale without any additional treatments in a fixed-bed setting using a standard TSA system. Further studies can focus on optimizing the mesopore volume of activated carbons to enhance their CO_2_ capture potential. Moreover, further investigation is required in order to assess the effect of other flue gas components, such as O_2_ and H_2_O, in order to explore the competition between reactions.

## Supplementary Material



## Notes

Pseudo second-order kinetic model for activated carbons at 30°C, for (a) 15% vol CO_2_ and (b) pure CO (Figure S1) (PDF)
