# Supplementary material for: Olive Mill Waste-Derived Activated Carbon for CO2 Capture Using Realistic Conditions
Source: Energy Fuels. 2025 Mar 7;39(11):5442–52. doi: 10.1021/acs.energyfuels.4c04880 (PMC12128171; doi:10.1021/acs.energyfuels.4c04880)
Supplement: Supplementary file 1 [file ef4c04880_si_001.pdf]

Olive mill waste derived activated carbon for CO_2 capture using realistic conditions

Pamela B. Ramos[1,2], Arminda Mamani[3], María F. Sardella[3], Amaya Arencibia[4], Raúl Sanz[5], Eloy S. Sanz-Pérez[4], *Marcela A. Bavio*[1,2], María Erans[6*]

[1]*Department of Chemical Engineering and Food Technology, Faculty of Engineering, UNCPBA, Avda. Del Valle 5737, B7400JWI, Olavarría, Buenos Aires, Argentina.*

[2]*INTELYMEC. CIFICEN (CICPBA-CONICET-UNCPBA), Avda. Del Valle 5737, B7400JWI, Olavarría, Buenos Aires, Argentina.*

[3]*Institute of Chemical Engineering, National University of San Juan-CONICET, San Juan Av. Libertador 1109 (west), 5400, San Juan, Argentina.*

[4]*Department of Chemical, Energy, and Mechanical Technology, ESCET, Universidad Rey Juan Carlos, C/Tulipán s/n, 28933 Móstoles, Madrid, Spain.*

[5]*Department of Chemical and Environmental Technology, ESCET, Universidad Rey Juan Carlos, C/Tulipán s/n, 28933 Móstoles, Madrid, Spain.*

[6]*Department of Chemical Engineering, Universidad de Valencia, Av. de la Universitat s/n, 46100 Burjasot, Valencia, Spain.*

**maria.erans@uv.es*

Number of pages: 2

Number of Figures: 1

Number of Tables: 2

Supplementary Information

SI-1. Kinetic models and parameters

The PFO and PSO models are expressed by equations (SI-2.1) and (SI-2.2).

$$\frac{dq_t}{dt} = k_1 \cdot (q_e - q_t) \qquad \textit{Eq. SI-1.1}$$

$$\frac{dq_t}{dt} = k_2 \cdot (q_e - q_t)^2 \qquad \textit{Eq. SI-1.2}$$

Where q_e and q_t represent the CO_2 adsorption at equilibrium and time (t), respectively; their units are mmol g^{-1}. k_1 and k_2 are the adsorption rate constant of PFO and PSO; with their units being min^{-1} and mmol g^{-1} min^{-1}, respectively.

The accuracy of the fit of the PFO and PSO kinetic models is analyzed by calculating R^2 and Dq (%), where R^2 is the coefficient of correlation and Dq is a function of the normalized standard deviation.

$$\Delta q\ (\%) = \sqrt{\frac{\sum\left(\frac{q_{exp}-q_{mod}}{q_{exp}}\right)^2}{N-1}} \times 100\% \qquad \textit{Eq. SI-1.3}$$

Where q_{exp} and q_m are the adsorption capacity of CO_2 experimentally and obtained by the model for N (numbers of data set points).

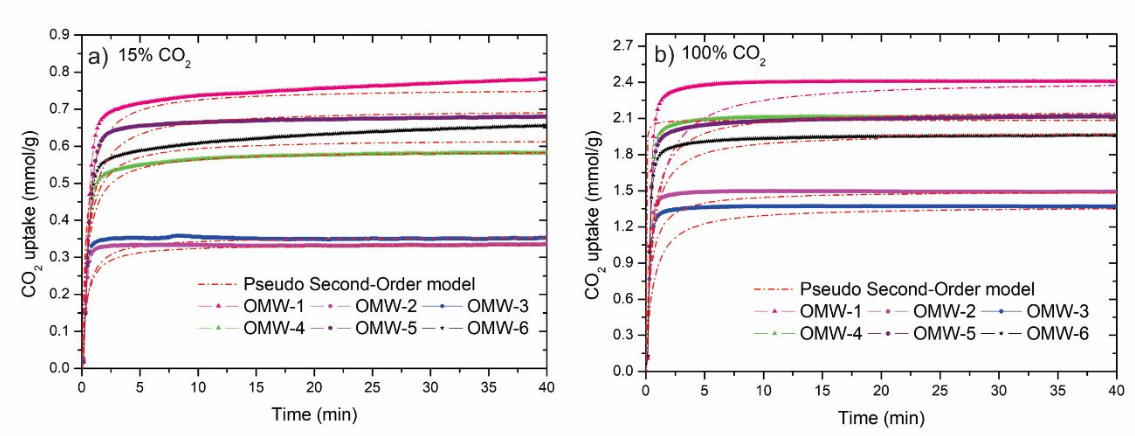

Figure SI-1: Pseudo second-order kinetic model for activated carbons at 30°C, for a) 15% vol CO_2 and b) pure CO_2

Table SI-1.1: Kinetic parameters of different chemically activated carbons for 15% vol CO_2 at 30°C.

Activated carbons	Kinetic model: Pseudo-first order (PFO) Parameters				
	qexp (mmol/g)	qmod (mmol/g)	kF (1/min)	R^2	Dq (%)
**OMW-1**	**0.846**	**0.789**	**0.153**	**0.9892**	**0.231**
**OMW-2**	**0.368**	**0.341**	**0.299**	**0.9929**	**0.229**
**OMW-3**	**0.386**	**0.356**	**0.349**	**0.9977**	**0.252**
**OMW-4**	**0.583**	**0.585**	**0.162**	**0.9996**	**0.107**
**OMW-5**	**0.678**	**0.659**	**0.159**	**0.9914**	**0.088**
**OMW-6**	**0.694**	**0.628**	**0.132**	**0.9902**	**0.229**
Activated carbons	Kinetic model: Pseudo-second order (PSO) Parameters				
	qexp (mmol/g)	qmod (mmol/g)	kS (g/mmol·min)	R^2	Dq (%)
OMW-1	0.846	0.836	0.09	0.8005	0.271
OMW-2	0.368	0.335	0.178	0.9121	0.300
OMW-3	0.386	0.355	0.163	0.9544	0.263
OMW-4	0.583	0.587	0.085	0.9547	0.213
OMW-5	0.678	0.697	0.061	0.9777	0.791
OMW-6	0.694	0.643	0.095	0.8977	0.242

Table SI-1.2: Kinetic parameters of different chemically activated carbons for 100% CO2 at 30°C.

Activated carbons	Kinetic model: Pseudo-first order (PFO) Parameters				
	qexp (mmol/g)	qmod (mmol/g)	kF (1/min)	R2	Dq (%)
**OMW-1**	**2.408**	**2.354**	**0.282**	**0.9948**	**2.31**
**OMW-2**	**1.496**	**1.512**	**0.311**	**0.9904**	**1.57**
**OMW-3**	**1.357**	**1.371**	**0.306**	**0.9990**	**1.11**
**OMW-4**	**2.125**	**2.124**	**0.281**	**0.9956**	**1.07**
**OMW-5**	**2.145**	**2.052**	**0.244**	**0.9992**	**3.87**
**OMW-6**	**2.402**	**2.350**	**0.257**	**0.9955**	**1.01**
Activated carbons	Kinetic model: Pseudo-second order (PSO) Parameters				
	qexp (mmol/g)	qmod (mmol/g)	kS (g/mmol·min)	R2	Dq (%)
OMW-1	2.408	2.401	0.090	0.8311	10.4
OMW-2	1.496	1.498	0.178	0.9552	9.1
OMW-3	1.357	1.365	0.163	0.9547	16.3
OMW-4	2.125	2.084	0.085	0.8749	13.7
OMW-5	2.145	2.165	0.061	0.9120	10.6
OMW-6	2.402	1.972	0.095	0.9259	9.9
